# Double Morphology of Co_9_S_8_ Coated by N, S Co-doped Carbon as Efficient Anode Materials for Sodium-Ion Batteries

**DOI:** 10.1186/s11671-020-3256-8

**Published:** 2020-01-22

**Authors:** Xuzi Zhang, Chaoqun Shang, Xin Wang, Guofu Zhou

**Affiliations:** 1 National Center for International Research on Green Optoelectronics, South China Academy of Advanced Optoelectronics, South China Normal University, Guangzhou, 51000 People’s Republic of China; 2 International Academy of Optoelectronics at Zhaoqing, South China Normal University, Zhaoqing, 526000 People’s Republic of China

**Keywords:** Co_9_S_8_, N, S co-doping, Rate performance, Node materials

## Abstract

Co_9_S_8_ is a potential anode material for its high sodium storage performance, easy accessibility, and thermostability. However, the volume expansion is a great hindrance to its development. Herein, a composite containing Co_9_S_8_ nanofibers and hollow Co_9_S_8_ nanospheres with N, S co-doped carbon layer (Co_9_S_8_@NSC) is successfully synthesized through a facile solvothermal process and a high-temperature carbonization. Ascribed to the carbon coating and the large specific surface area, severe volume stress can be effectively alleviated. In particular, with N and S heteroatoms introduced into the carbon layer, which is conducive to the Na^+^ adsorption and diffusion on the carbon surface, Co_9_S_8_@NSC can perform more capacitive sodium storage mechanism. As a result, the electrode can exhibit a favorable reversible capacity of 226 mA h g^−1^ at 5 A g^−1^ and a favorable capacity retention of 83.1% at 1 A g^−1^ after 800 cycles. The unique design provides an innovative thought for enhancing the sodium storage performance.

## Introduction

With the rapid development of power reserve systems in electric vehicles and portable electronic products, sodium-ion batteries (SIBs) have become a strong competitor to lithium-ion batteries (LIBs), because of the similar charge-discharge behavior to LIBs, low cost, and vast natural reserves [1–3]. And the electrochemical potential of Na (− 2.71 V vs the standard hydrogen electrode, SHE) is higher than that of Li (− 3.04 V) with 330 mV, which makes SIBs possible to meet large-scale energy storage demands [4–6]. However, the most important challenge in SIBs is the large volume expansion during the process of sodiation originated from the great strain derived from the larger radius of Na^+^ (1.02 Å) than Li^+^ (0.76 Å) [7, 8]. This will result in severe pulverization and exfoliation of active materials from copper foil and further lead to poor cycling performance. Therefore, rational design of anode materials is an impending concern.

Diverse anode materials have been reported for their high theoretical capacity, such as transition-metal sulfides (TMSs) [9–14], transition-metal oxides (TMOs) [15–18], phosphides [19–22], and carbon composites [23–26]. Among them, cobalt-based MSs (such as CoS, CoS_2_, Co_3_S_4_, and Co_9_S_8_) have attracted great attention for its near-metallic conductivity and easy accessibility [27–29]. Specifically, cubic Co_9_S_8_ attracts much attention for its great thermostability. Unfortunately, it is still impeded by the severe volume variation, slow Na^+^ diffusion rate, and poor conductivity [30–32]. Great efforts have been made to handle the shortcomings of Co_9_S_8_.

So far, most studies have also focused on designing novel carbon materials with heteroatom (N, P, S, B) doped, such as sandwich-like structures with N, S-doped RG O[33, 34], nanoflower-like N-C/CoS 2[35], Co_9_S_8_ coated with N-doped carbon nanospheres [36, 37], and N, S-doped nanofibers [38, 39]. Carbon coating can not only enhance the conductivity of TMSs, but also remit the stress stemming from the volume expansion. In particular, with the heteroatom doping, the electronic structure of carbon can be modified to improve the physical and chemical properties by generating extrinsic defects, expanding the interlayer distance and offering additional electron transfer route when heteroatoms are bonded with carbon atoms [40–44].

Herein, we synthesize double morphology of Co_9_S_8_, containing nanofibers and hollow nanospheres, both coated with N, S co-doped carbon (denoted as Co_9_S_8_@NSC), for highly stable SIBs. Nanofiber-like structure provides long-range continuous electron transport, while the hollow nanospheres enhance the infiltration of electrolyte. The N, S co-doped carbon layer can provide more free electrons, which benefit the adsorption of Na^+^ on the surface and enhance the integral conductivity. Due to the hardness of carbon coating and 3D network, volume variation during sodium ion insertion/extraction can be well alleviated from atomic and 3D level. And the high specific surface area can enhance the capacity of pseudo-capacitance contribution, leading to excellent rate performance. As a result, SIBs with Co_9_S_8_@NSC can deliver a stable capacity retention of 318 mA h g^−1^ after 800 cycles at 1 A g^−1^ with the coulombic efficiency of ~ 100%, making it a promising anode for large-scale SIBs.

## Experimental Methods

### Synthesis of Co_9_S_8_@NSC

In a typical process, the composites were prepared by coaxial electrospinning following alcohol-thermal method and carbonization.

### Preparation of Electrospun Nanofibers

0.74 g PAN (Sigma-Aldrich, MW = 150,000) and 9 ml DMF was stirred for overnight to form the homogeneous outer solution, while 1.8 g Cobalt(II) acetylacetonate (Co(acac)_2_, Aladdin, purity ≥ 99%) and 0.74 g PAN (Macklin, MW = 150000) were mixed with 9 ml DMF and stirred at the same time as the dark red inner solution. Then, the two kinds of solutions were conducted by coaxial electrospinning (needle size: inner 17 G, outer:22 G). The distance between the needle and Al foil collector was 15 cm, and the condition temperature was maintained at 65 °C. Then, electrical potential was applied at 15 kV with flow rate of two syringes both at 1.5 ml h^−1^. The final precursor fibers were dried at 60 °C in vacuum for 24 h.

### Sulfuration and Carbonization

The obtained nanofibers were firstly mixed with 50 ml ethanol containing thioacetamide (TAA, Aladdin, purity ≥ 99%) in 100 ml Teflon-lined stainless-steel autoclave at 120 °C for 6 h by solvothermal method. The final product was obtained by carbonizing at 700 °C for 1 h with a heating rate of 5 °C min^−1^ and cooling down naturally. For comparison, sample without cobalt (N, S co-doped carbon, denoted as NSC) was also prepared using the same method mentioned above without adding Co(acac)_2_.

### Structural Characterization

The morphology and structure of the Co_9_S_8_@NSC were characterized by scanning electron microscopy (SEM, ZEISS Gemini 500) and transmission electron microscopy (TEM, JEM-2100HR). Thermal gravity analysis (TGA) test was performed to evaluate the content of Co_9_S_8_ by Netzsch STA449. The crystalline structures and surface valence state analyses were detected by X-ray photoelectron spectroscopy (XPS, ESCALAB 250Xi), X-ray powder diffraction (XRD, Bruker D8 Advance), and Raman spectra. The specific surface area and pore size distribution were recorded from the Brunauer-Emmett-Teller (BET, Micromeritics ASAP-2020) analysis instrument.

### Electrochemical Measurements

Slurries were obtained by mixing active materials, poly (vinylidene fluoride) (PVDF), and Super P (weight ratio of 8:1:1) with N-methylpyrrolidone (NMP). Then, the working electrode was prepared by coating the slurries uniformly on a precut copper foil (diameter 12 mm) and dried at 60 °C in air and vacuum overnight, respectively. The CR2032-type coin cells were assembled with sodium metal as the reference electrode, glass fiber membrane as the separator, and the as-prepared copper foil as the anode. The electrolyte was 1 M NaClO_4_ in EC/DMC (EC:DMC = 1:1, in volume) with 5.0% FEC. The assembly procedures were all carried out in an Ar-filled glove box (O_2_ < 0.1 ppm, H_2_O < 0.1 ppm). The cyclic voltammetry (CV) and electrochemical impedance spectroscopy (EIS) results were obtained from an electrochemical workstation (CHI660E, Shanghai Chen Hua Instruments Ltd). And the galvanostatic discharge-charge tests were conducted in a NEWARE battery testing system.

## Results and Discussion

**Fig. 1:**
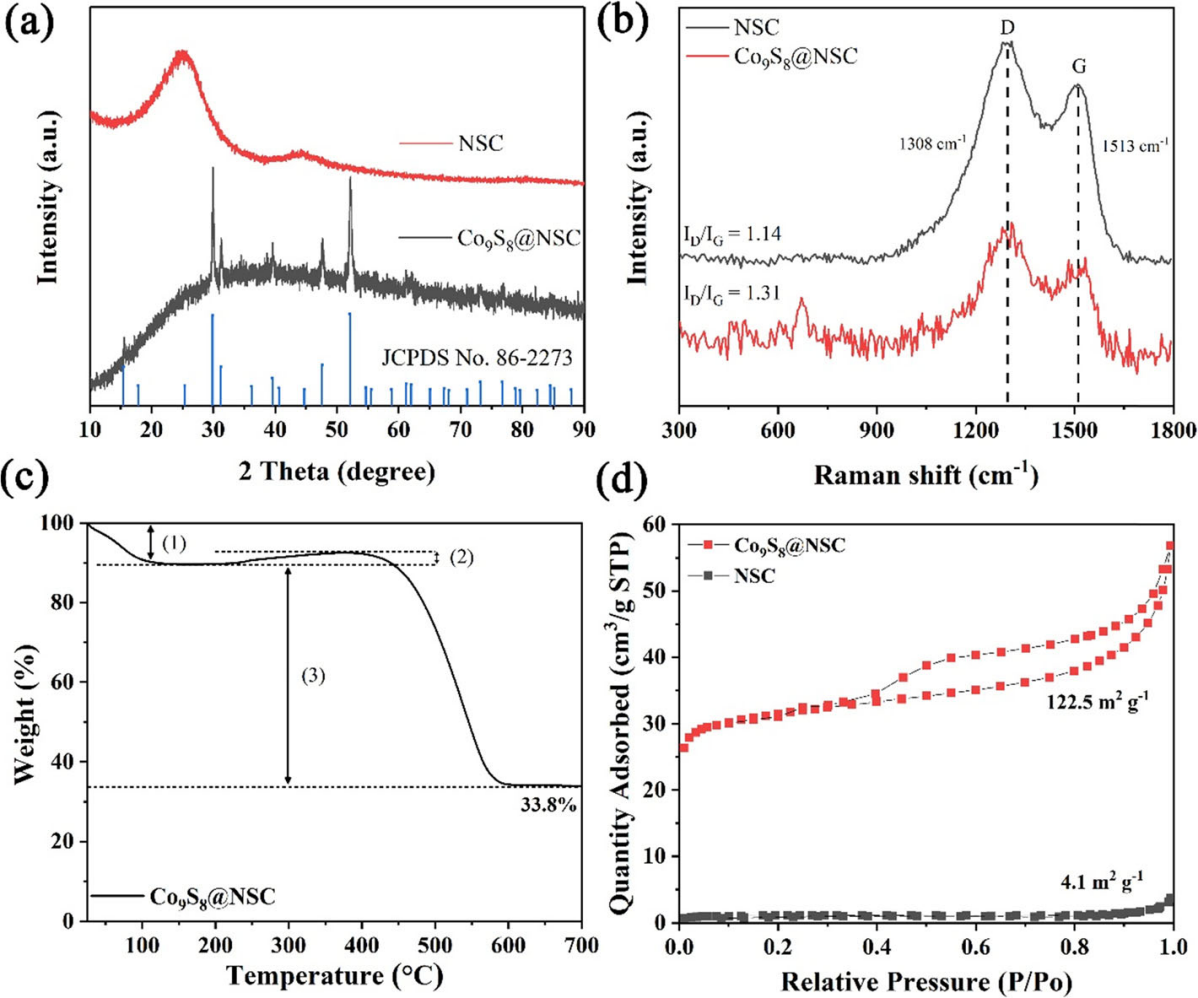
**a** X-ray diffraction (XRD) of Co_9_S_8_@NSC and NSC. **b** Raman spectra of Co_9_S_8_@NSC and NSC. **c** TG of Co_9_S_8_@NSC. **d** N_2_ adsorption and desorption isotherms of Co_9_S_8_@NSC and NSC

The synthesis process of Co_9_S_8_@NSC is illustrated in Additional file 1: Scheme S1, including coaxial electrospinning, solvothermal sulfuration, and carbonization. The crystallinity of Co_9_S_8_@NSC and NSC after those procedures is shown in Fig. 1a. The XRD curve of Co_9_S_8_@NSC displays typical characteristic diffraction peaks in accord with the cubic Co_9_S_8_ phase (JCPDS no. 86-2273), while NSC only exhibits peaks of hard carbon. The broad peak at 24.8° is corresponding to (111) plane of amorphous carbon. Strikingly, it is lower than the standard value of 26.6°, indicating expanded interlayer distance and lower graphitization derived from N, S co-doped sites into the carbon [41]. The Raman spectra (Fig. 1b) also confirms the existence and composition of carbon in the composites. Co_9_S_8_@NSC and NSC both exhibit two obvious peaks of 1308 cm^−1^ and 1513 cm^−1^, representing the D band and G band of carbon, respectively. Besides, Co_9_S_8_@NSC owns a weak typical peak located at 671 cm^−1^, corresponding to the Co_9_S_8_. In detail, the D band is attributed to the structure defects of amorphous carbon, while the G band is due to the *E*_*2g*_ vibration mode of Sp^2^ bond between graphitic carbon atoms [45]. The slightly larger I_*D*_/I_*G*_ of Co_9_S_8_@NSC (1.31) than NSC (1.14) indicates more defects exist in the composites, resulted from N doping and S doping.

The weight ratio of Co_9_S_8_ is measured by thermogravimetric analysis (TGA) as shown in Fig. 1c. After heating to 700 °C in air with 10 °C min^−1^, multistep reaction is involved with the final product as Co_3_O_4_, which can be ascribed to: (1) the evaporation of trace water (below 100 °C), (2) the oxidation of Co_9_S_8_ to CoSO_*x*_ (from 200 to 400 °C), and (3) the decomposition of carbon and continuous oxidation of CoSO_*x*_ (above 400 °C). According to these reactions, the content of Co_9_S_8_ in the composites is calculated as 40.1%. Co_9_S_8_@NSC exhibits typical type IV isotherm curves, indicating the mesoporous structure, while the NSC is microporous (Fig. 1d). The specific surface area of Co_9_S_8_@NSC (122.5 m^2^ g^−1^) is much larger than NSC (4.1 m^2^ g^−1^), which can benefit the infiltration of electrolyte for fast insertion/extraction of Na^+^ and remit the severe volume change. The pores of Co_9_S_8_@NSC with average size of 8.6 nm (shown in Additional file 1: Figure S1) are derived from the pyrolysis of chemical groups of polyacrylonitrile, dissolution of Co(acac)_2_ from the nanofibers, and the formation of hollow Co_9_S_8_ nanospheres.

**Fig. 2:**
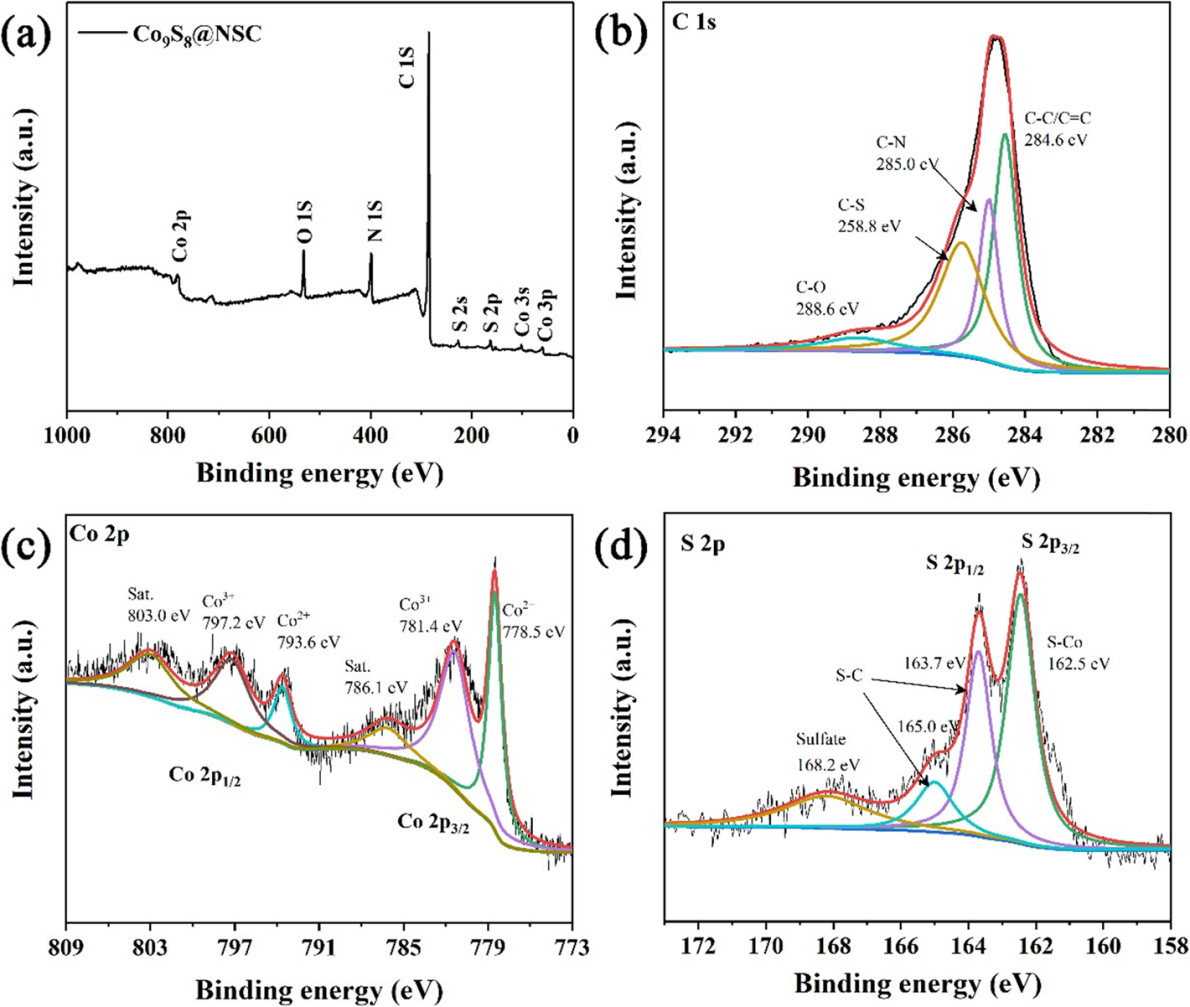
**a** XPS survey spectrum. **b** C 1s. **c** Co 2p, and **d** S 2p of Co_9_S_8_@NSC

X-ray photoelectron spectroscopy (XPS) is conducted to identify the chemical composition of Co_9_S_8_@NSC. The signals in the survey spectrum are accord with five elements of C, N, O, S, and Co in Fig. 2a. The presence of O should be due to the exposure of the sample in air with some oxygen adsorbed on the surface. The high-resolution spectrum of C 1s (Fig. 2b) displays four peaks, which are located at 284.6 eV (C-C/C=C), 285.0 eV (C-N), 285.8 eV (C-S), and 288.6 eV (C=N) [46]. The Co 2p spectrum was shown in Fig. 2c. Peaks located at 786.1 eV and 803 eV can be fitted to the satellite peaks of Co 2p_3/2_ and Co 2p_1/2_, respectively. In addition, peaks of 778.5 eV and 793.6 eV and another two peaks at 781.4 eV and 797.2 eV belong to Co^2+^ and Co^3+^, respectively [36]. Furthermore, the S 2p spectrum (Fig. 2d) is fitting into four peaks, consisting of 162.45 eV (S-Co), 163.7 eV (S-C), 165 eV (S-C), and 168.2 eV (sulfate) [47]. And N 1s spectra (Additional file 1: Figure S2) contain three peaks at 398.4 eV, 400.1 eV, and 401.0 eV, which correspond to pyridinic N, pyrrolic N, and graphitic N, respectively [48]. Compared to the alone carbon materials, introducing N,S co-doped carbon sites can provide more free electrons, which benefits the adsorption of Na^+^ on the surface and enhances the integral conductivity [41]. The results of all XPS spectrums confirm the presence of N, S co-doping in Co_9_S_8_@NSC.

**Fig. 3:**
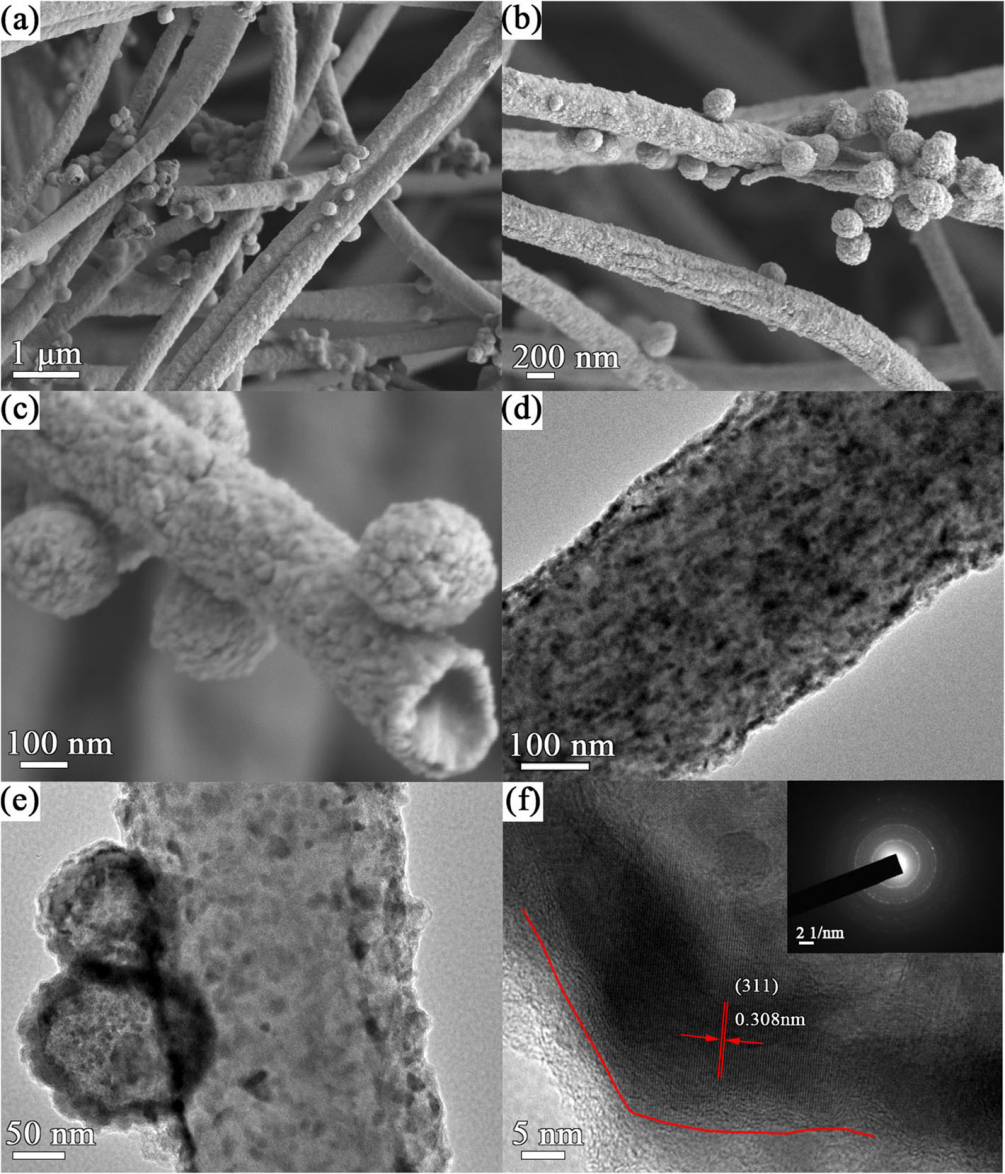
**a**–**c** SEM and **d**–**f** TEM of Co_9_S_8_@NSC at different magnification. (The inset of (**f**) is the SAED of Co_9_S_8_@NSC.)

The interesting morphologies and detailed internal structures of Co_9_S_8_@NSC and NSC are measured by SEM and TEM. Figure 3a–c depicts the Co_9_S_8_@NSC consist of two kinds of structure including hollow nanospheres and nanofibers. These nanofibers are composed by carbonization of PAN fibers. The formation of nanospheres adhered on the nanofibers may be attributed to the decomposition and sulfurization of Co(acac)_2_ which was dissolved from the inside of PAN electrospinning fibers. And the composites own the distinctly coarse surface, which can enhance the wettability of electrolyte. Additional file 1: Figure S3a–c displays the NSC morphology with only smooth nanofibers cemented to each other without nanospheres, which can be due to the absence of Co-based compound and melt of PAN nanofibers in the process of carbonization. The elemental composition is confirmed by EDS mapping (Additional file 1: Figure S4), with C, N, Co, and S elements uniformly distributing in the composites. And this further demonstrates the successful doping of N, S element.

As displayed in Fig. 3d–f, TEM images reveal the internal details of the morphologies of Co_9_S_8_@NSC. Figure 3d and e show the Co_9_S_8_ nanoparticles are embedded in carbon nanofibers and hollow nanospheres, which confirms the point proposed above about the formation of the hollow nanospheres. Figure 3f reveals the interplanar distance of 0.308 nm, matching well with the (311) planes of Co_9_S_8_, while the NSC in Additional file 1: Figure S3d–f manifests the traditional character of hard carbon. Co_9_S_8_ nanoparticles with size all bellowing 50 nm distribute uniformly in the composite, and the thickness of carbon coating layer is measured 3–5 nm (Fig. 3f). Owing to the rough surface, hollow structure, and carbon coating, severe pulverization and exfoliation of active materials resulting from volume variation might be alleviated effectively.

**Fig. 4:**
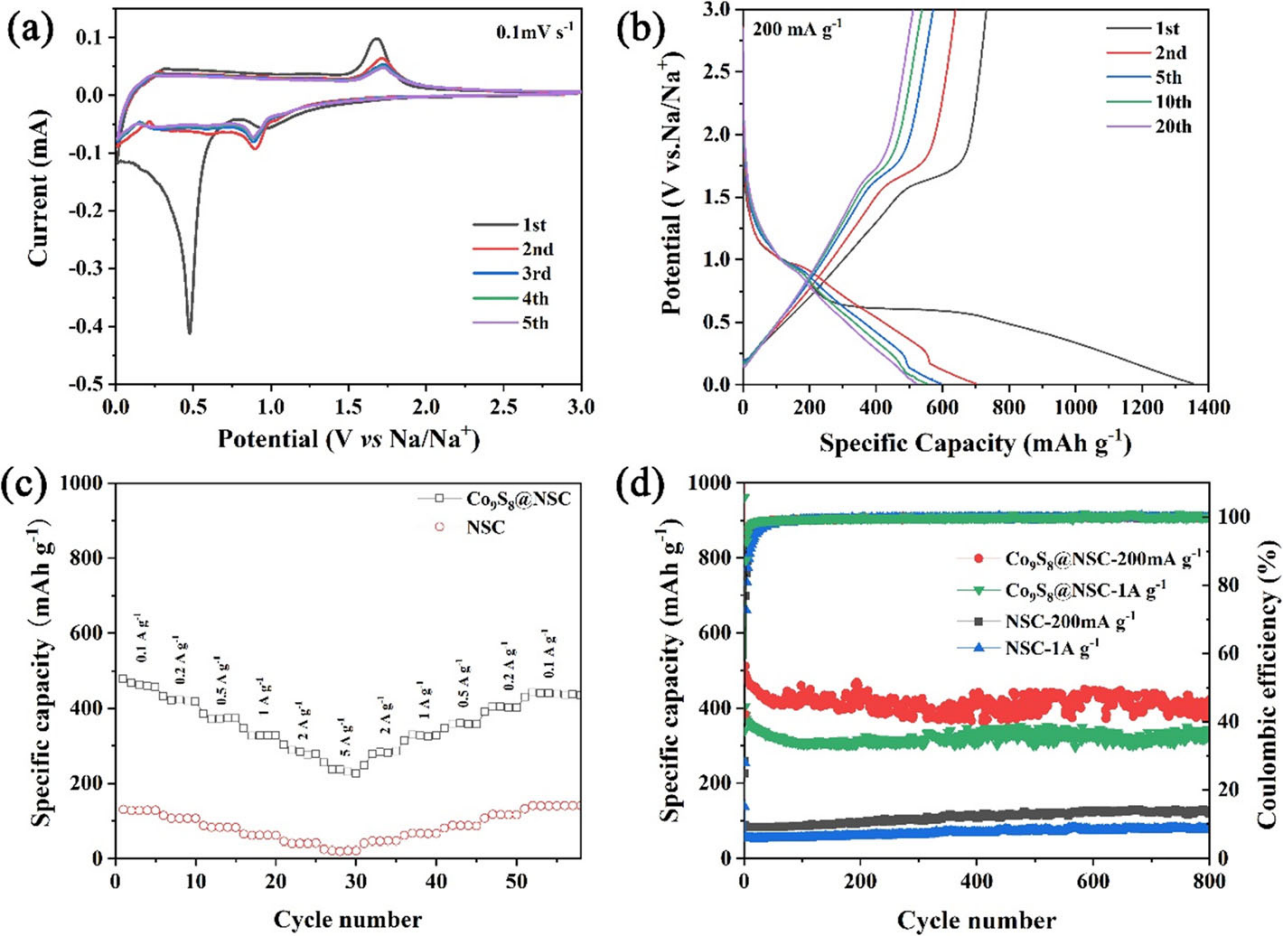
CV curves (**a**) and charge-discharge profiles (**b**) with different cycles of Co_9_S_8_@NSC. Rate capability (**c**) and cycling performance (**d**) of Co_9_S_8_@NSC compared with NSC

To investigate the electrochemical performance of Co_9_S_8_@NSC, 2032-type coin cells are assembled for electrochemical tests. As shown in Fig. 4a, the CV curves of Co_9_S_8_@NSC record the initial five cycles at a scan rate of 0.1 mV s^−1^. The first cycle is far different from the subsequent cycles with a broad peak at 0.476 V in cathodic sweep, which is assigned to the formation of solid electrolyte interface (SEI) film, irreversible intercalation of Na^+^, and the stepwise conversion of Co_9_S_8_ to Co and Na_2_S [49]. In the first anodic sweep, two oxidation peaks of 0.375 V and 1.682 V can be attributed to the multistep reaction of Co to CoS_x_ [36]. The following CV curves of Co_9_S_8_@NSC are gradually overlapped, indicating the high electrochemical reversibility. For comparison, CV curves of NSC in Additional file 1: Figure S5a show the typical characteristic peaks of carbon, which represents the adsorption and insertion of Na^+^ in carbon nanofibers. Figure 4b and Additional file 1: Figure S5b display the charge/discharge curves for different cycles of Co_9_S_8_@NSC and NSC with the initial coulombic efficiency (CE) of 54.1% and 28.3%, respectively. The relatively low CE is caused by the irreversible formation of SEI film and electrolyte consumption [7]. The curves of these two samples manifest distinctive voltage platform of Co_9_S_8_ and carbon, which are in accord with the results of CV tests (Fig. 4a and Additional file 1: Figure S5a).

Rate performance is shown in Fig. 4c. The capacity of Co_9_S_8_@NSC can reach 226 mAh g^−1^ at 5 A g^−1^, while NSC can only maintain 21 mAh g^−1^. And then, when the current density recovers to 100 mAh g^−1^, the capacity can be well returned to 440 mAh g^−1^. Figure 4d shows the cycling performance of Co_9_S_8_@NSC. The discharge capacities of Co_9_S_8_@NSC maintain at 423 mAh g^−1^ at 200 mA g^−1^ and 318 mAh g^−1^ at 1 A g^−1^ after 800 cycles with initial coulombic efficiency at 42.3% and 37.4%, respectively. This can be explained by the reaction mechanism transformation from intercalation/deintercalation at low current density to adsorption/desorption of Na^+^ at high current density [2]. Apparently, the capacities of Co_9_S_8_@NSC are all higher than those of NSC, which is due to the more active sites derived from Co_9_S_8_ and N, S co-doping. In addition, the capacity retention of 87.4% at 200 mA g^−1^ and 83.1% at 1 A g^−1^ after 800 cycles indicate the stable cycling performance and benign reaction reversibility of Co_9_S_8_@NSC.

To understand the interfacial properties and internal resistances of Co_9_S_8_@NSC and NSC, electrochemical impedance spectra (EIS) has been conducted. As presented in Additional file 1: Figure S6a, the Nyquist plots of Co_9_S_8_@NSC after different cycles show typical semicircles in high-frequency region (charge transfer resistance, *R*_ct_) and an oblique line in low-frequency region (Warburg resistance, *W*). Before the initial cycle, the largest *R*_ct_ (about 1600 Ω) of Co_9_S_8_@NSC is caused by insufficient infiltration of electrolyte. After 5 cycles, the *R*_ct_ becomes very small as 153 Ω for the formation of SEI film and well contact with electrolyte. Furthermore, the decrease of the *R*_ct_ can be also attributed to the process of activation of the Co_9_S_8_@NSC electrode interfacial. After 10 cycles, it almost keeps the same value, which indicates the excellent stability. For comparison, EIS of NSC is also investigated in Additional file 1: Figure S6b–e. The initial *R*_ct_ of NSC is smaller than Co_9_S_8_@NSC, indicating the higher electroconductivity of NSC. As the cycle is going on, the *R*_ct_ of Co_9_S_8_@NSC gradually becomes smaller than that of NSC, owing to the larger specific surface area and enough infiltration of electrolyte. These results mentioned above support the benign cycling and rate performance of Co_9_S_8_@NSC.

**Fig. 5:**
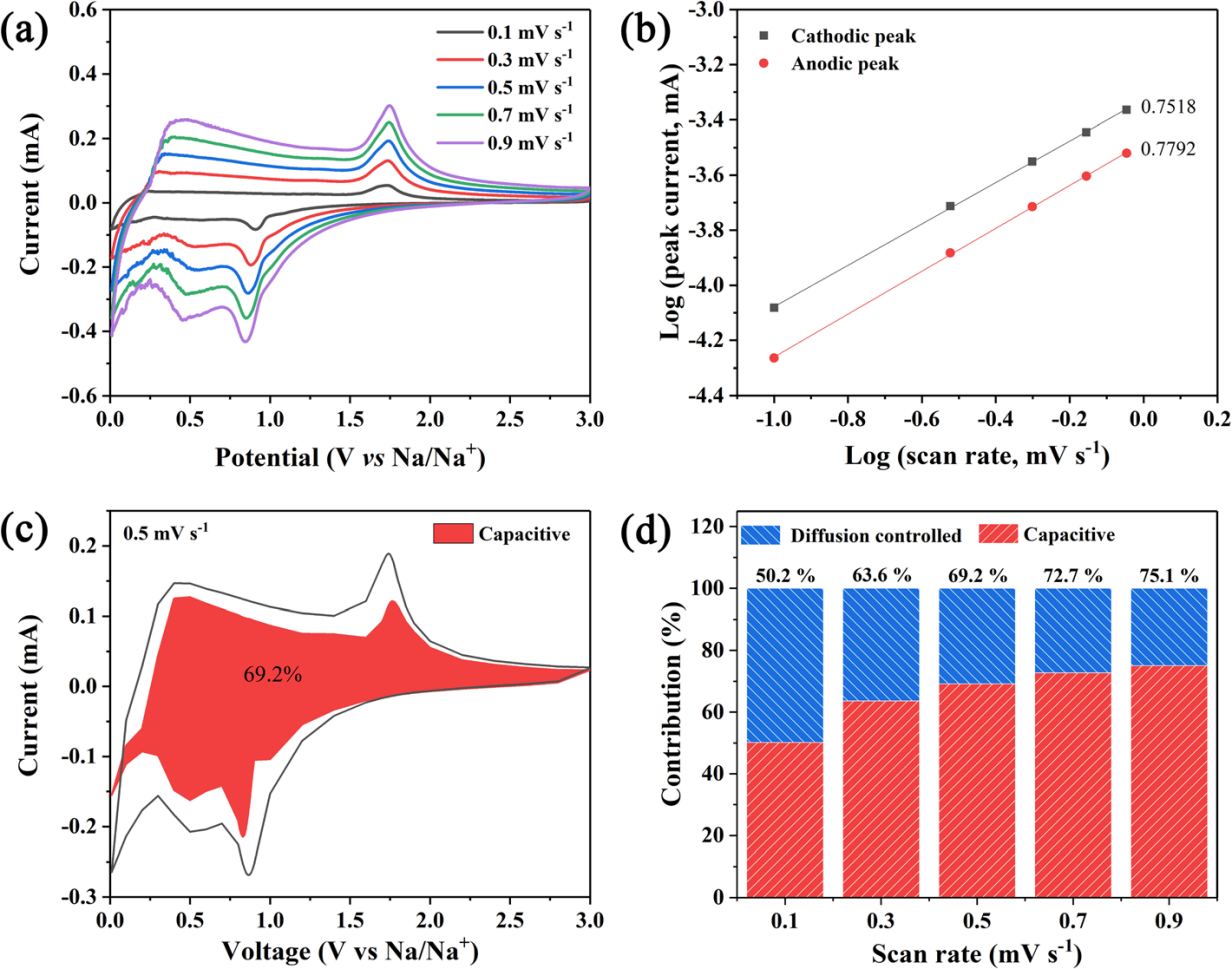
**a** CV curves of Co_9_S_8_@NSC at various scan rates. **b** The linear relationship between log (*v*) and log (*I*). **c** Capacitive contribution (red) in CV curve. **d** Contribution ratio of capacitive capacity at different scan rates

To gain further insight into the electrochemistry of Co_9_S_8_@NSC electrode, a kinetic analysis is conducted. The CV curves at different sweep rates from 0.1 to 0.9 mV s^−1^ are collected and shown in Fig. 5a. Varying from common ionic diffusion, the peak current (*I*, mA) is not completely linearly dependent to *v*^1/2^ (*v* is the scan rate, mV s^−1^), indicating the coexistence of non-faradic and faradic behaviors [35, 50]. And the results can be verified by the relation between log(*I*) and log(*v*), according to equation of log(*I*) = *b* log(*v*) + log(*a*). When the value of *b* reaches 0.5 or 1, it indicates that the reaction mechanism is totally controlled by ionic diffusion or capacitive behavior, respectively [51, 52]. As shown in Fig. 5b, the calculated values of *b* are 0.7518 (cathodic peak) and 0.7792 (anodic peak), which means more capacitive behavior.

Furthermore, the contribution of capacitive behavior can be obtained from the equation: *i*(V) = *k*_1_*v* + *k*_2_*v*^1/2^ [42], where *i*(V) is the current at a fixed voltage, *v* is the sweep rate, and *k*_1_*v* and *k*_2_*v*^1/2^ represent the capacitive capacity and ion diffusion capacity, respectively. When sweep rate is 0.5 mV s^−1^, the contribution ratio of the capacitive capacity reaches 69.2% (Fig. 5c). For different sweep rates, Fig. 5d illustrates an obvious tendency of the capacitive capacity ratio increasing with the scan rate from 0.1 to 0.9 mV s^−1^. The increasing capacitive contribution can be ascribed to high specific area and abundant active sites, which furthermore may be responsible for the excellent rate performance of the Co_9_S_8_@NSC electrode. All these results reveal a fast kinetic of Co_9_S_8_@NSC which resulted from the capacitive effect.

According to Fig. 5a, the Na^+^ diffusion coefficients (*D*_*Na+*_) can be estimated from the strongest peak current (*I*_*p*_) and sweep rates (*v*) by Randles-Sevick equation [32]:

$$ {I}_p=2.69\times {10}^5{n}^{3/2}A{D}_{Na+}^{1/2}{v}^{1/2}C $$

where *n*, *A*, and *C* represent the number of transferred electrons in the process of Na^+^ intercalation/deintercalation, the surface area, and the molar concentration of Na^+^, respectively. The *D*_*Na*+_ of Co_9_S_8_@NSC is proportional to the slope of the linear relations between *I*_p_ and *v*^1/2^ (Additional file 1: Figure S7). As a result, the slope values of anodic peak and cathodic peak of Co_9_S_8_@NSC are much positive and negative than those of NSC, respectively, which means that the *D*_*Na*+_ of Co_9_S_8_@NSC is much higher than NSC in the process of Na^+^ intercalation/deintercalation. In detail, it can be ascribed to favorable infiltration of electrolyte and much exposed active sites derived from the larger specific surface area of Co_9_S_8_@NSC.

## Conclusions

In summary, a novel double morphology of Co_9_S_8_, containing hollow nanospheres and nanofibers, with coating N, S co-doped carbon layer has been successfully synthesized using coaxial electrospinning following sulfurization by solvothermal method and carbonization. Owing to the larger specific surface area and carbon coating, Co_9_S_8_@NSC can accommodate the volume change during the charge/discharge process. More uniformly, active sites derived from Co_9_S_8_ and N, S co-doped position can not only contact with much more electrolyte, but also accelerate diffusion of Na^+^ and reversible reaction between Na^+^ and Co_9_S_8_@NSC. When applied as anode materials, Co_9_S_8_@NSC can deliver a high reversible specific capacity of 318 mAh g^−1^ after 800 cycles at 1 A g^−1^ with the coulombic efficiencies remaining almost 100%, while the large surface area and abundant N, S co-doped sites can lead to the excellent rate capability. The study offers more possibilities of cobalt sulfides in designing effective anode materials for SIBs.

## Supplementary information

**Additional file 1: Scheme S1.** Preparation process of Co_9_S_8_@NSC. **Figure S1.** BJH pore width distribution of Co_9_S_8_@NSC (a) and NSC (b). **Figure S2.**  N 1 s XPS spectra of Co_9_S_8_@NSC. **Figure S3.** (a-c) SEM and (d-f) TEM of NSC at different magnification. (The inset of (f) is the SAED of NSC.) **Figure S4.** EDS mapping of Co_9_S_8_@NSC. **Figure S5.** (a) CV curves of NSC with different cycles at 0.1 mV s^-1^; (b) Charge-discharge profiles of NSC at various cycles at 200 mA g^-1^. **Figure S6.** (a) Nyquist plots of Co_9_S_8_@NSC and (b) NSC before and after cycles; EIS curves comparison between Co_9_S_8_@NSC and NSC for initial (c), 5 cycles later (d) and 10 cycles later (e). **Figure S7.** The linear relation between *I*_*p*_ and *v*^1/2^ according to the Randles-Sevick equation.

## Data Availability

All data used within this manuscript are available upon request.

## Abbreviations

BET: Brunauer-Emmett-Teller analysis
CE: Coulombic efficiency
Co(acac)_2_: Cobalt(II) acetylacetonate hydrate
CV: Cyclic voltammetry
DMC: Dimethyl carbonate
EC: Ethylene carbonate
EIS: Electrochemical impedance spectroscopy
LIBs: Lithium-ion batteries
NSC: N, S co-doped carbon
SIBs: Sodium-ion batteries
TGA: Thermal gravity analysis
XRD: X-ray diffraction
