# Supplementary material for: Double Morphology of Co9S8 Coated by N, S Co-doped Carbon as Efficient Anode Materials for Sodium-Ion Batteries
Source: Nanoscale Res Lett. 2020 Jan 22;15:19. doi: 10.1186/s11671-020-3256-8 (PMC6975880; doi:10.1186/s11671-020-3256-8)
Supplement: Supplementary file 1 — Additional file 1: Scheme S1. Preparation process of Co9S8@NSC. Figure S1. BJH pore width distribution of Co9S8@NSC (a) and NSC (b). Figure S2. N 1 s XPS spectra of Co9S8@NSC. Figure S3. (a-c) SEM and (d-f) TEM of NSC at different magnification. (The inset of (f) is the SAED of NSC.) Figure S4. EDS mapping of Co9S8@NSC. Figure S5. (a) CV curves of NSC with different cycles at 0.1 mV s-1; (b) Charge-discharge profiles of NSC at various cycles at 200 mA g-1. Figure S6. (a) Nyquist plots of Co9S8@NSC and (b) NSC before and after cycles; EIS curves comparison between Co9S8@NSC and NSC for initial (c), 5 cycles later (d) and 10 cycles later (e). Figure S7. The linear relation between Ip and v1/2 according to the Randles-Sevick equation. [file 11671_2020_3256_MOESM1_ESM.docx]

**Supporting Information**

**Double morphology of Co_9_S_8_ coated by N, S co-doped carbon as efficient anode materials for sodium-ion batteries**

Xuzi Zhang^1^, Chaoqun Shang ^1,2^*, Xin Wang^1,2^*, Guofu Zhou^1,2^

* Correspondence: chaoqun.shang@ecs-scnu.org, wangxin@scnu.edu.cn

^1^ National Center for International Research on Green Optoelectronics, South China Normal University, Guangzhou 51000, P. R. China

^2^ International Academy of Optoelectronics at Zhaoqing, South China Normal University, Zhaoqing 526000, P. R. China


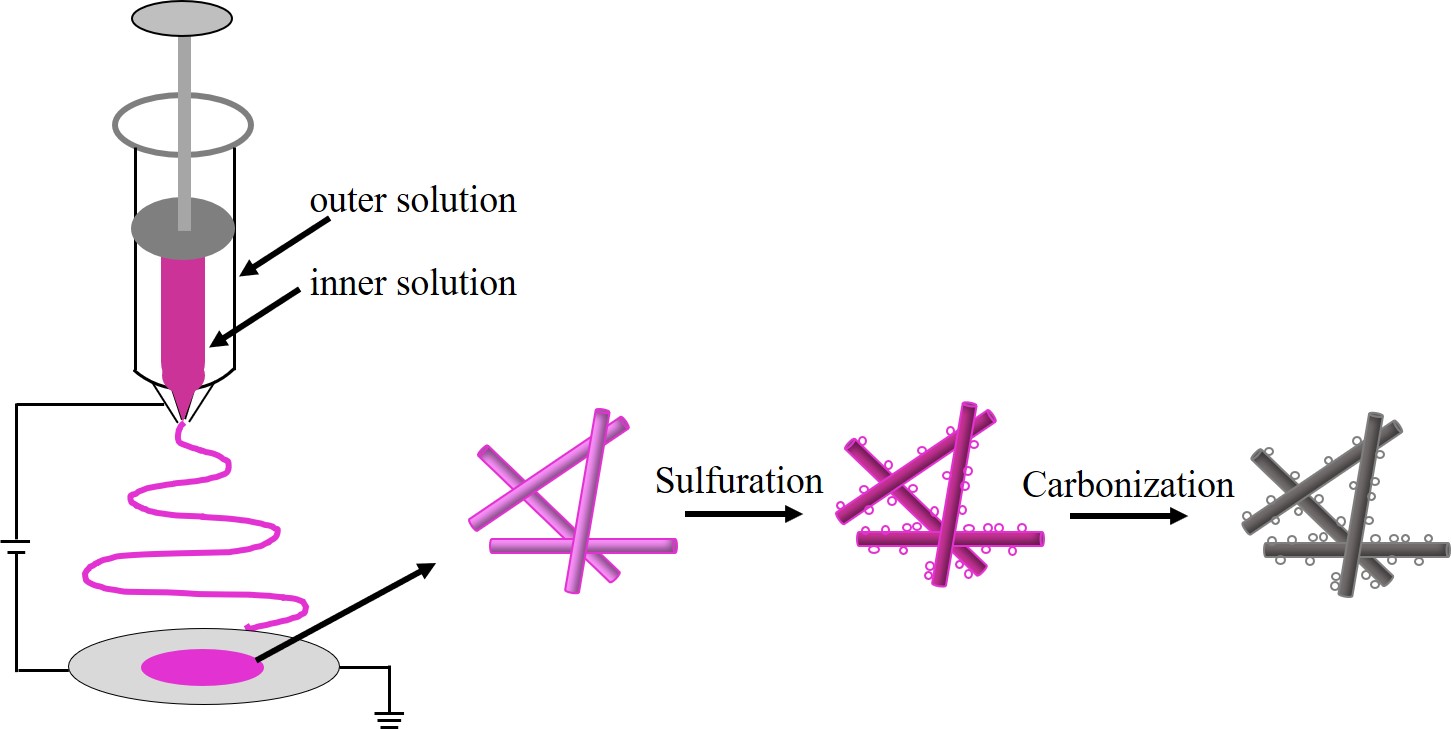


**Scheme S1.** Preparation process of Co_9_S_8_@NSC


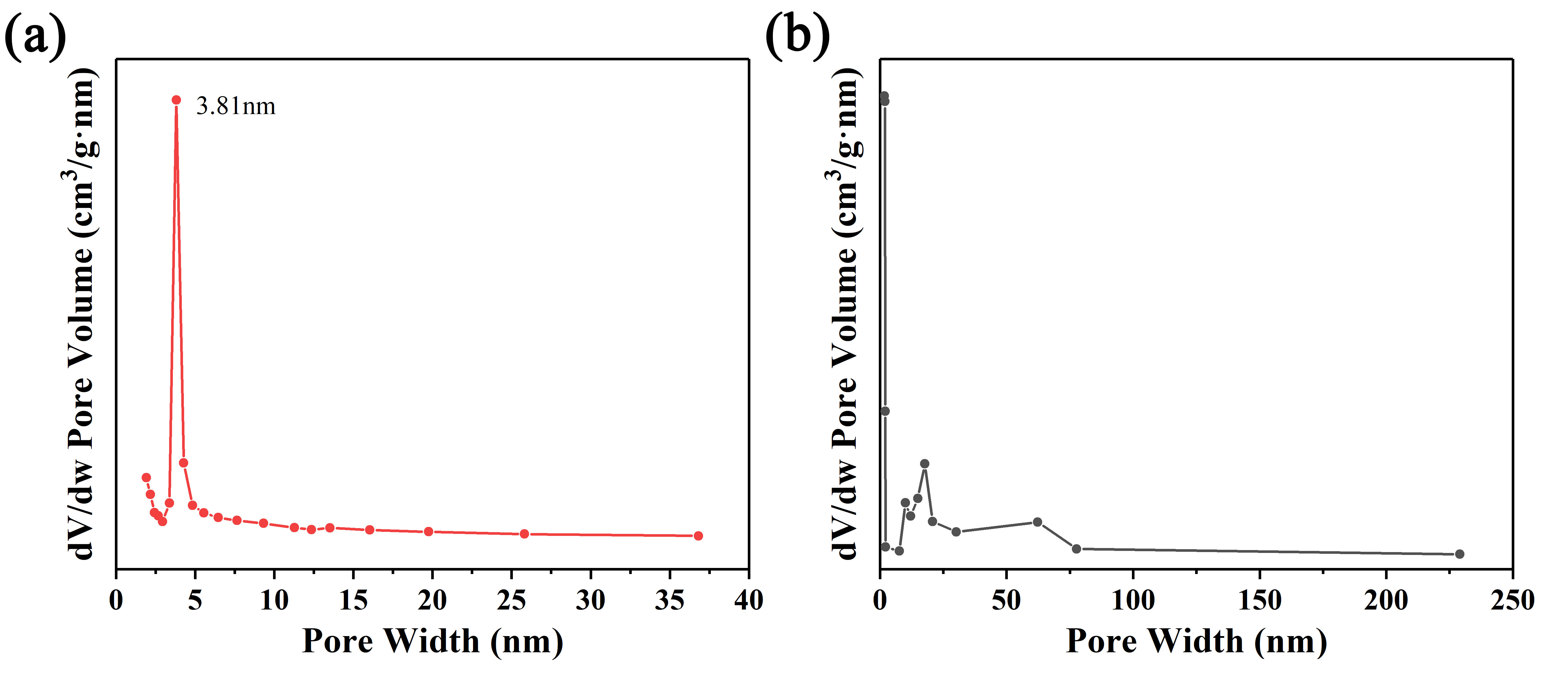


**Fig. S1** BJH pore width distribution of Co_9_S_8_@NSC (a) and NSC (b).


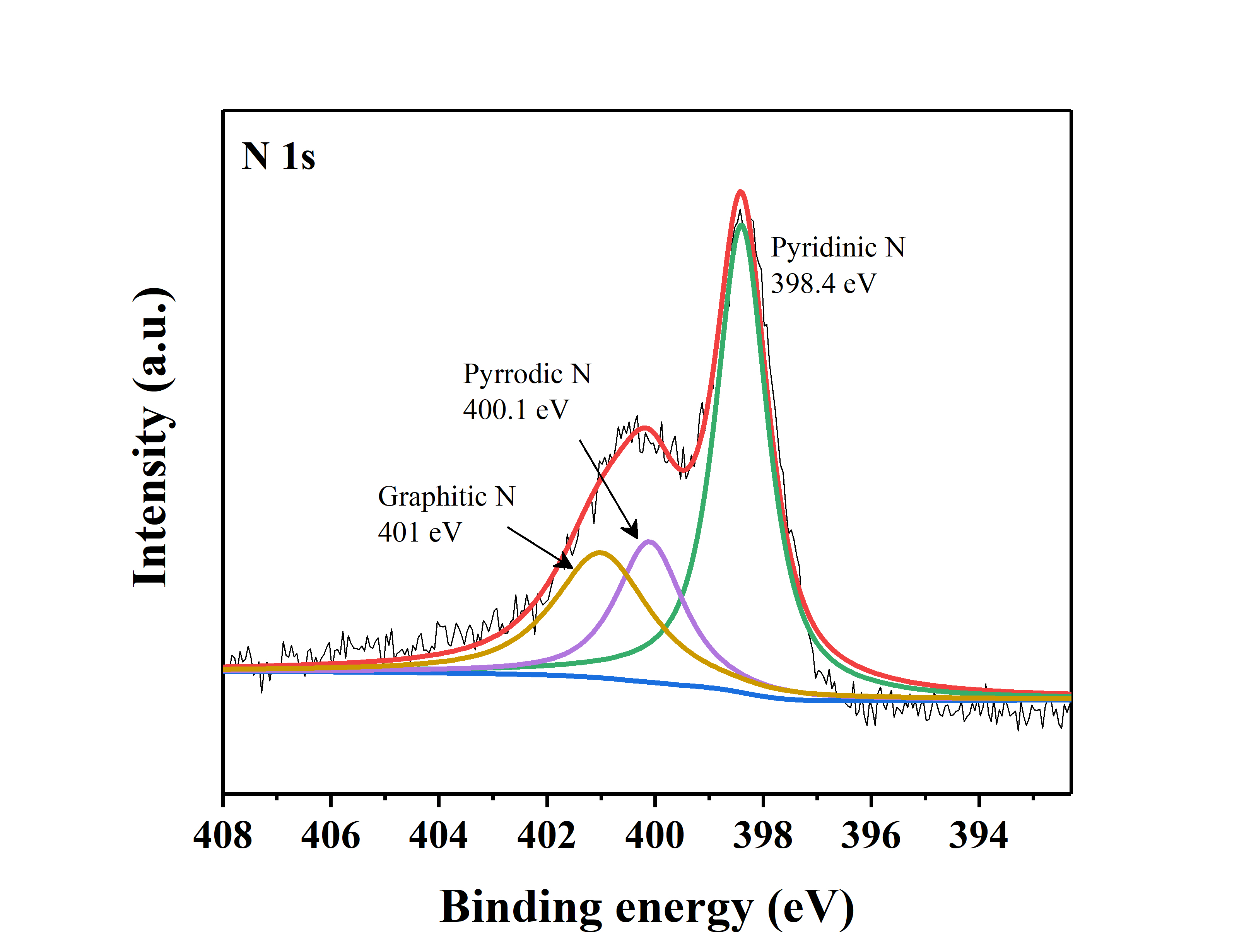


**Fig. S2** N 1s XPS spectra of Co_9_S_8_@NSC.


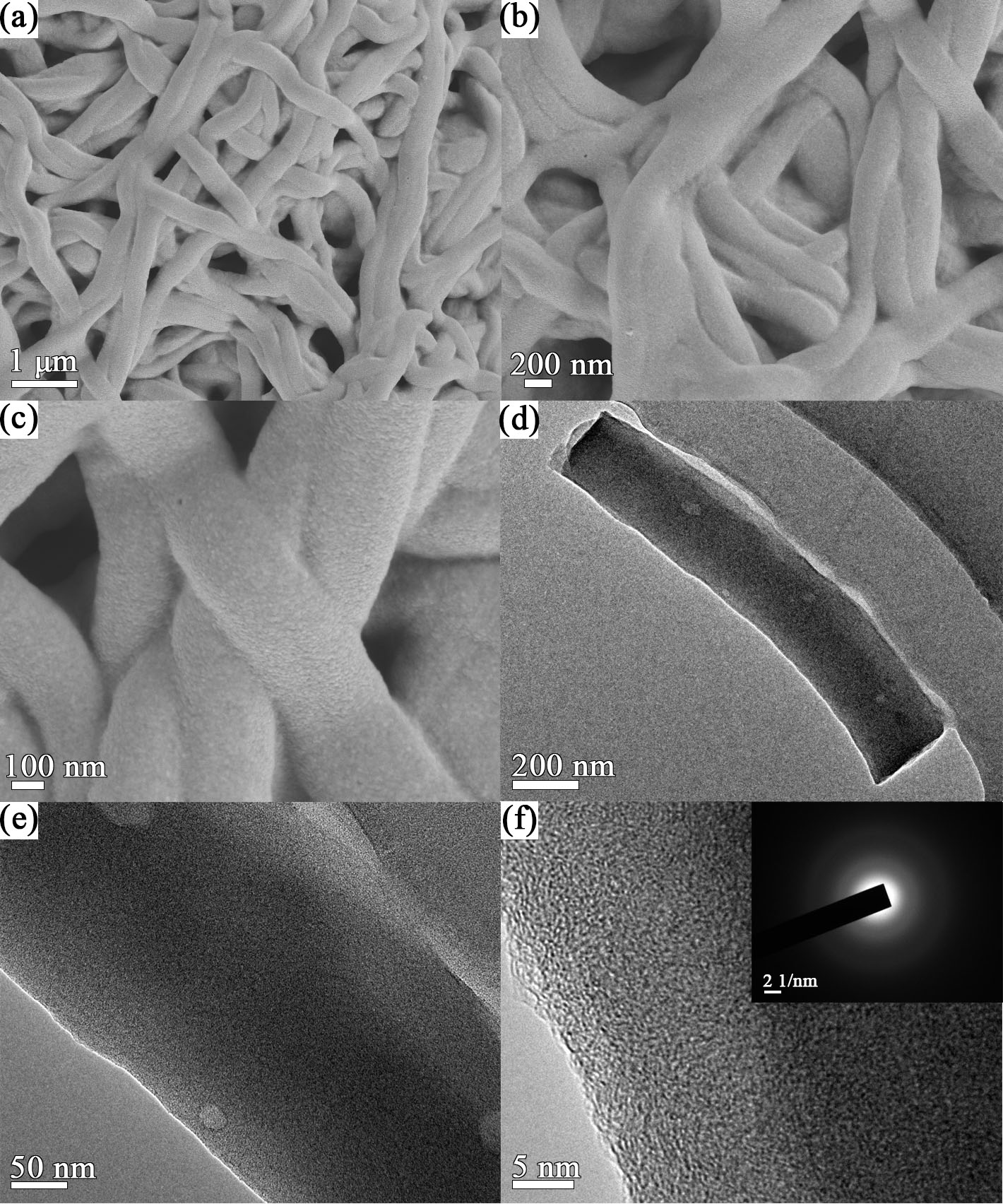


**Fig. S3** (a-c) SEM and (d-f) TEM of NSC at different magnification. (The inset of (f) is the SAED of NSC.)


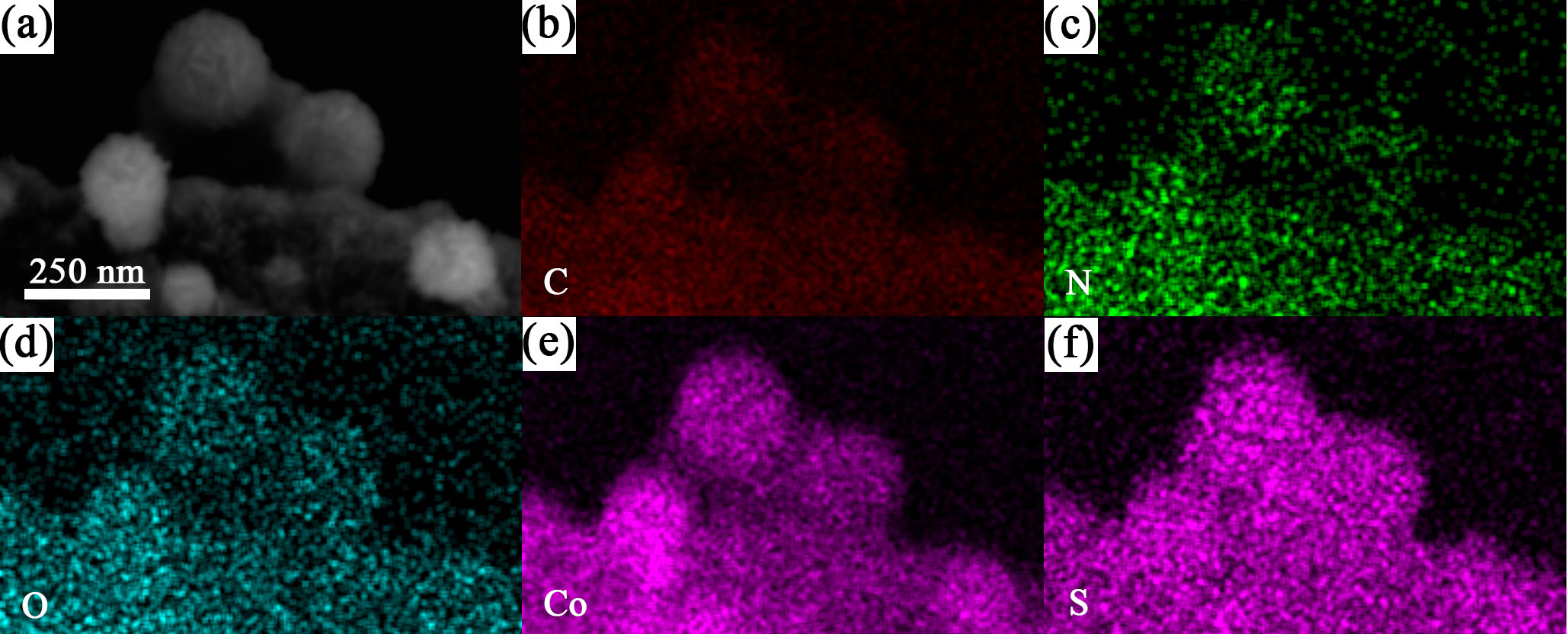


**Fig. S4** EDS mapping of Co_9_S_8_@NSC.


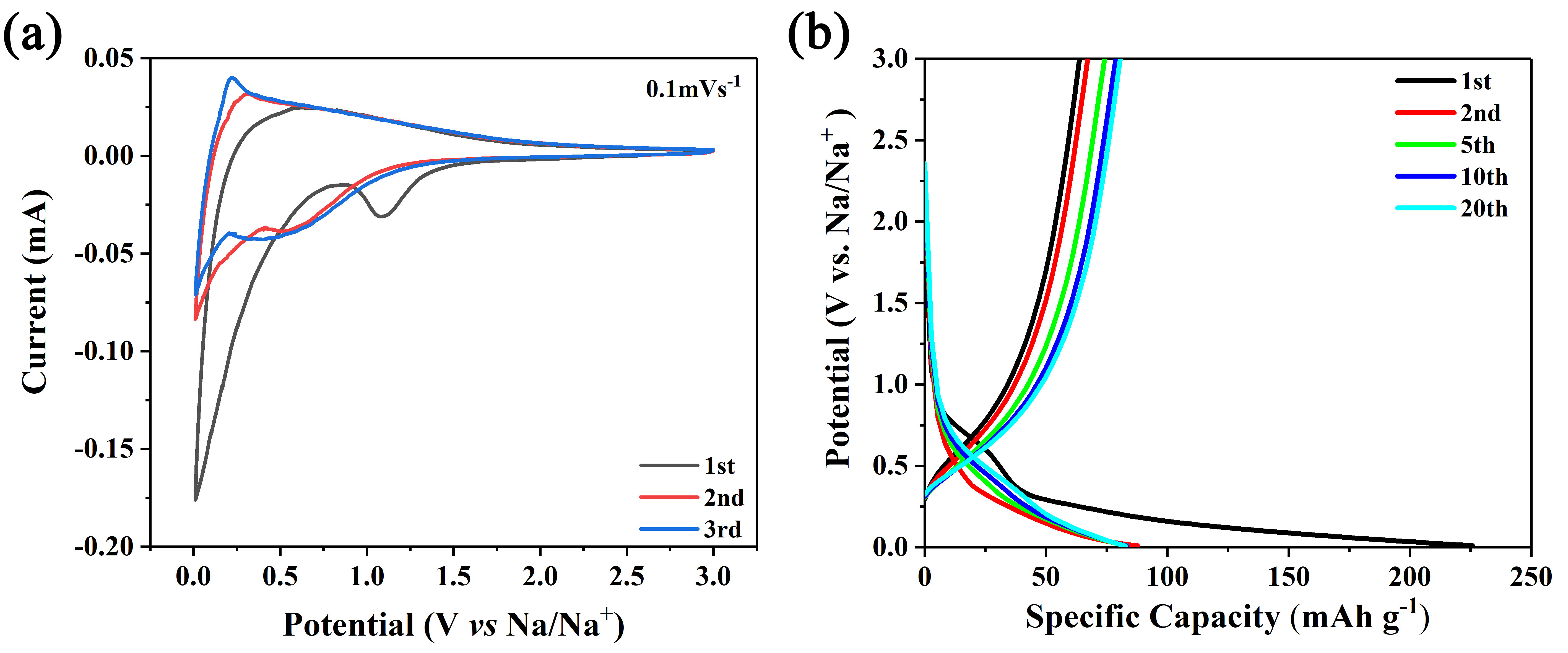


**Fig. S5** (a) CV curves of NSC with different cycles at 0.1 mV s^-1^; (b) Charge-discharge profiles of NSC at various cycles at 200 mA g^-1^.


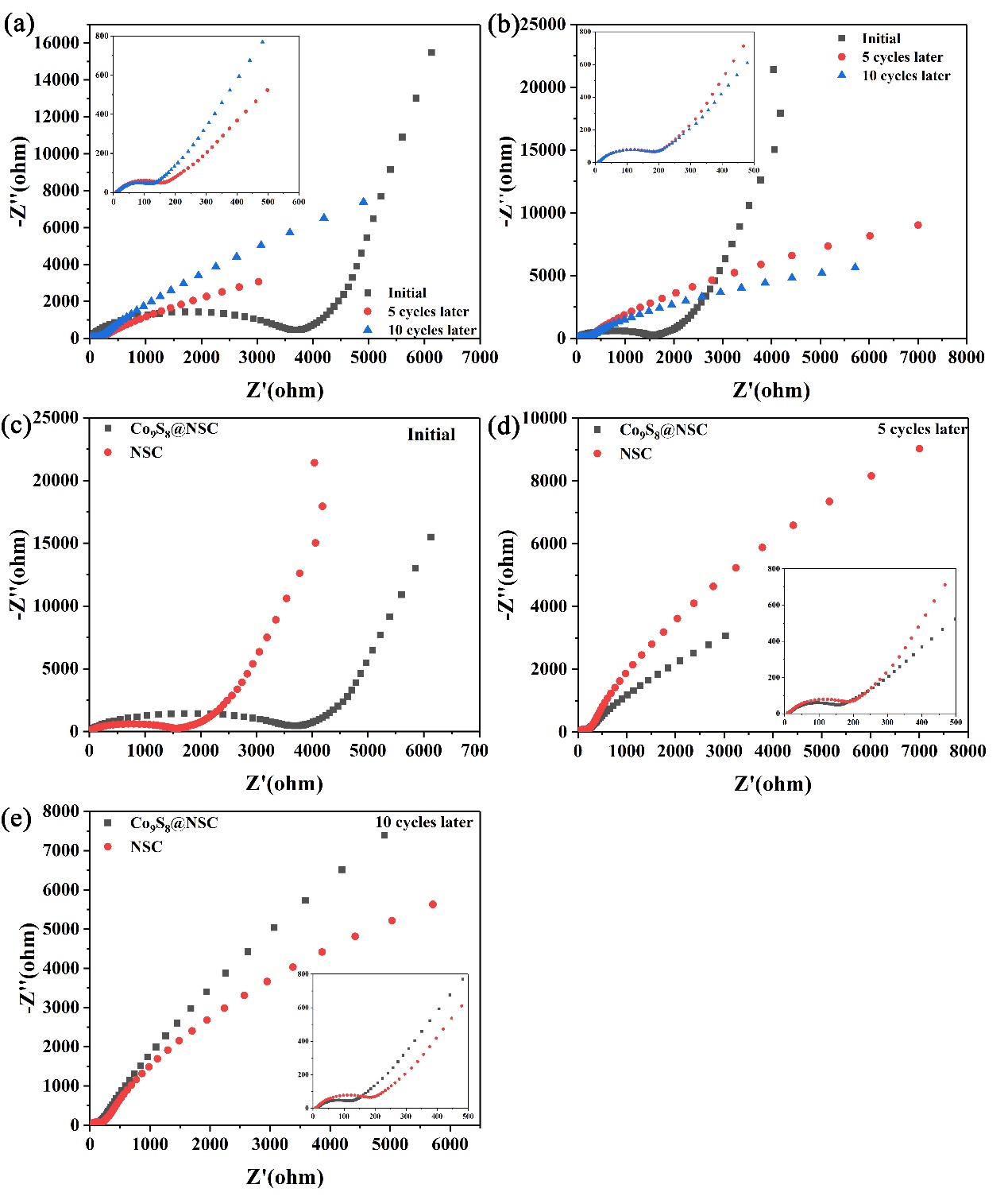


**Fig. S6** (a) Nyquist plots of Co_9_S_8_@NSC and (b) NSC before and after cycles; EIS curves comparison between Co_9_S_8_@NSC and NSC for initial (c), 5 cycles later (d) and 10 cycles later (e).


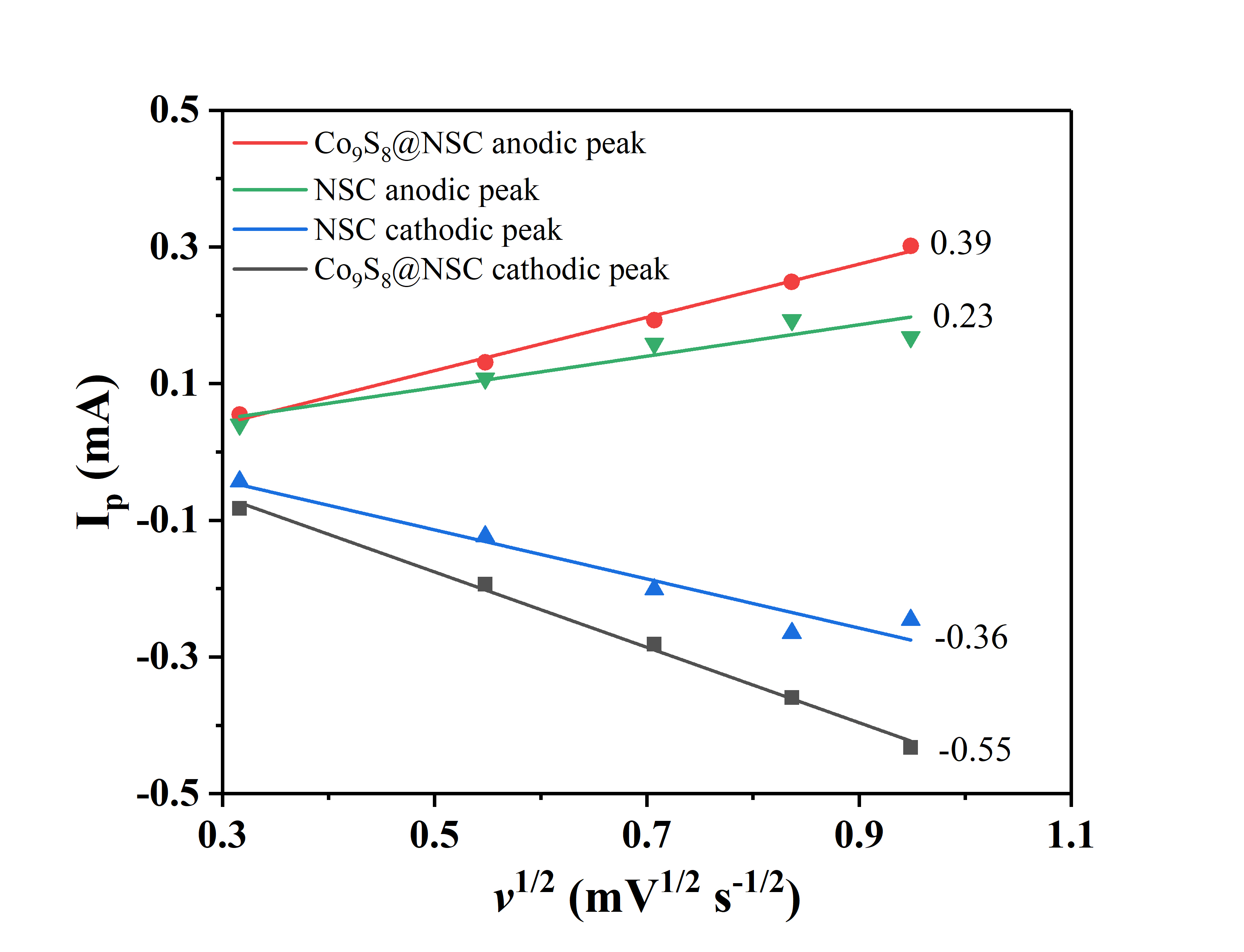


**Fig. S7** The linear relation between *I_p_* and *v*^1/2^ according to the Randles-Sevick equation.
